# Effect of Using an Indoor Air Quality Sensor on Perceptions of and Behaviors Toward Air Pollution (Pittsburgh Empowerment Library Study): Online Survey and Interviews

**DOI:** 10.2196/mhealth.8273

**Published:** 2018-03-08

**Authors:** Gabrielle Wong-Parodi, M Beatrice Dias, Michael Taylor

**Affiliations:** ^1^ Carnegie Mellon University Pittsburgh, PA United States; ^2^ Arviz, Inc Pittsburgh, PA United States

**Keywords:** indoor air pollution, particulate matter, inhalation, decision aids

## Abstract

**Background:**

Air quality affects us all and is a rapidly growing concern in the 21st century. We spend the majority of our lives indoors and can be exposed to a number of pollutants smaller than 2.5 microns (particulate matter, PM_2.5_) resulting in detrimental health effects. Indoor air quality sensors have the potential to provide people with the information they need to understand their risk and take steps to reduce their exposure. One such sensor is the Speck sensor developed at the Community Robotics, Education and Technology Empowerment Lab at Carnegie Mellon University. This sensor provides users with continuous real-time and historical PM_2.5_ information, a Web-based platform where people can track their PM_2.5_ levels over time and learn about ways to reduce their exposure, and a venue (blog post) for the user community to exchange information. Little is known about how the use of such monitors affects people’s knowledge, attitudes, and behaviors with respect to indoor air pollution.

**Objective:**

The aim of this study was to assess whether using the sensor changes what people know and do about indoor air pollution.

**Methods:**

We conducted 2 studies. In the first study, we recruited 276 Pittsburgh residents online and through local branches of the Carnegie Library of Pittsburgh, where the Speck sensor was made available by the researchers in the library catalog. Participants completed a 10- to 15-min survey on air pollution knowledge (its health impact, sources, and mitigation options), perceptions of indoor air quality, confidence in mitigation, current behaviors toward air quality, and personal empowerment and creativity in the spring and summer of 2016. In our second study, we surveyed 26 Pittsburgh residents in summer 2016 who checked out the Speck sensor for 3 weeks on the same measures assessed in the first study, with additional questions about the perception and use of the sensor. Follow-up interviews were conducted with a subset of those who used the Speck sensor.

**Results:**

A series of paired t tests found participants were significantly more knowledgeable (t_25_=−2.61, *P*=.02), reported having significantly better indoor air quality (t_25_=−5.20, *P*<.001), and felt more confident about knowing how to mitigate their risk (t_25_=−1.87, *P*=.07) after using the Speck sensor than before. McNemar test showed participants tended to take more action to reduce indoor air pollution after using the sensor (χ^2^_25_=2.7, *P*=.10). Qualitative analysis suggested possible ripple effects of use, including encouraging family and friends to learn about indoor air pollution.

**Conclusions:**

Providing people with low- or no-cost portable indoor air quality monitors, with a supporting Web-based platform that offers information about how to reduce risk, can help people better express perceptions and adopt behaviors commensurate with the risks they face. Thus, thoughtfully designed and deployed personal sensing devices can help empower people to take steps to reduce their risk.

## Introduction

Air quality affects all of us and is a rapidly growing concern in the 21st century [1]. According to results of a recent study, environmental exposures such as air pollution may even be linked to autism spectrum disorder rates among children [2]. Furthermore, airborne particulates smaller than 2.5 microns (particulate matter, PM_2.5_) can cause significant harm to human health because they not only lodge deep in the lungs but also cross the air-blood barrier into the human bloodstream and endocrine systems. Exposure to PM_2.5_ has been associated with asthma attacks, respiratory disease, arrhythmia, and cardiovascular disease [2].

### Air Pollution

Pittsburgh, in particular, has a long history of pollution stemming from coal mining and other industrial activities [3]. Although the city is now notably cleaner, there are still many invisible and visible pollutants contaminating the air we breathe [4,5]. According to a 2012 report from the Pennsylvania Department of Health, among Pennsylvania’s 67 counties, Pittsburgh’s Allegheny County had the 6th highest number of emergency room visits caused by asthma (21 visits per 10,000 residents) [5,6]. Moreover, during the 2008-2009 school year, 12.1% of Allegheny County students were reportedly diagnosed with asthma. These rates are alarming and also have significant economic impact for the community, with each asthma-related hospital stay (from 2008-2010) costing over US $20,000 on average [6]. Conversely, improving air quality in Pittsburgh could yield substantial economic benefits. In a 2013 report, RAND Corporation estimated that reducing the city’s 2012 levels of PM_2.5_ to meet National Ambient Air Quality Standards yields approximately US $488 in economic value [7]. These findings were driven primarily by reductions in premature mortality among residents and provide evidence that there may be considerable economic benefits associated with reducing residents’ exposure to PM_2.5_.

Although outdoor air pollution is widely accepted as a problem, indoor air quality can often be overlooked because the level of visible pollution indoors is relatively low. Indoor air pollution can be caused by outdoor contaminants seeping in through windows or poor air filtration systems, or generated from indoor sources such as smoking, cooking, and vacuuming. Many of these sources can produce PM_2.5_ inside our homes, schools, and offices, but because these particles are so small (about a 30th of the diameter of a human hair or less), they would be invisible to us, except in very high concentrations. We spend the majority of our lifetime in indoor spaces, so our level of exposure to these indoor pollutants can be very high. However, unlike outdoor air pollution, which is a significant challenge to mitigate and requires years to enact necessary air quality regulations, indoor air quality can be managed by anyone.

### Risk Perceptions and Behaviors

Although individuals are better able to control their air quality indoors, whether or not individuals or families take action to reduce their risk of exposure to pollution indoors is largely dependent on how they perceive this risk [8,9]. One key requisite for this risk perception is the awareness that there is a risk [10]. Moreover, providing people with personalized information about their risk influences attitudes and behaviors more powerfully than simply informing them about the risk in general [11-13]. Furthermore, research in the domain of risk perceptions has found that people use experiential/affective processes to understand risk [14] and that helping people experience that risk may help them better learn about it [15].

By contrast, studies in other domains, notably health, have often found that fear and worry can undermine individuals’ resolve to act, unless they see opportunities for effective action [16]. An illustrative early study by Leventhal et al found that arousing concern about tetanus increased more favorable attitudes and intentions to get a vaccination, but people rarely followed through and actually received one [17]. However, when the researchers augmented their fear appeal with a specific plan, a map with instructions on how to get to the clinic, they found people actually followed through on their intent to get vaccinated. Indeed, the most effective fear appeals are those coupled with high-efficacy messages showing effective measures that people can take to reduce their risk [18]. One way to help people make the connection between their activities and lifestyle choices, subsequent changes in PM_2.5_ concentration levels, and ways they can mitigate their risk is through the introduction of indoor air quality monitors.

### Personal Sensor Technologies for Indoor Air Pollution

**Figure 1:**
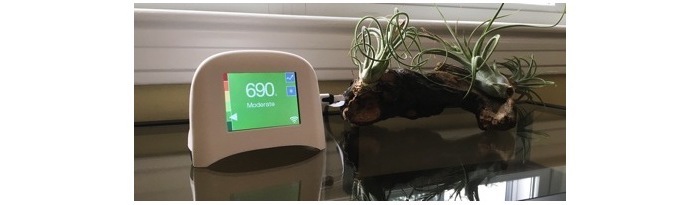
Image of the Speck monitor home screen displaying air quality reading.

There are a growing number of personal sensor technologies, including those that detect ambient PM_2.5_ levels, available in the market [19]. A few of these technologies are portable, allowing people to place the monitor in different places in their home and conduct a variety of activities such as cooking and vacuuming to see how their indoor air quality is affected by reading directly off the monitor. Some also offer a companion mobile phone app, which may provide continuous real-time and historical PM_2.5_ information. Other sensors, such as the Speck (Figure 1 [20]), developed at the Community Robotics, Education and Technology Empowerment Lab at Carnegie Mellon University, also have a Web-based platform where people can track their PM_2.5_ levels over time and learn about ways to reduce their exposure; these sensors also provide a venue (blog post) for the user community to exchange information.

Little research has been conducted to evaluate the effect of these types of monitors on people’s knowledge, attitudes, and behaviors; however, one study investigating the use of air quality visualizations over a 4-week period with 14 participants did find changes in attitudes and an increase in prohealth behaviors [21,22]. Research findings in the area of personal wearable fitness devices on physical activities are mixed, with some studies finding an increase in activity [23,24] and others finding no change [25]. Therefore, whether and how indoor air quality monitors influence people’s behavior to improve indoor air quality remains an empirical question.

Our research objective was to assess whether and how the use of a Speck sensor to monitor indoor air quality empowers people to reduce their risk of exposure to indoor pollutants. To that end, we conducted 2 studies. Our first exploratory study gathers baseline information about what Pittsburgh residents, recruited online or from their local library, generally know and do about indoor air pollution; how confident they are that those actions are effective; and how they would want to learn about it (eg, are indoor air quality sensors appropriate?). Our second study evaluates the effect of using a sensor to monitor indoor air pollution on what people know and do about indoor air pollution among those library patrons from our exploratory first study. These patrons were invited to use the monitor for a period of up to 3 weeks, with their views and behaviors being surveyed after using the monitor. To better explicate our findings, we also interviewed a select subset of those who checked out the monitor from their public library.

## Methods

### Study 1: Baseline Views and Behaviors of the General Public

#### Survey Protocol

After a brief introduction to the study, eligible participants (18 years or older, and living in Pittsburgh) took a 10- to 15-min survey to assess their views and behaviors related to indoor air pollution, and basic demographics.

#### Variables

##### Knowledge

Knowledge of air pollution was assessed by asking, “How much do you know about air quality?” where 1=none and 5=everything.

##### Health

The seriousness of perceived health consequences was assessed by asking, “Do you think air quality can cause or make worse the following issues?” Participants were encouraged to check all of the potential issues from a list of 8 items, which included asthma and other respiratory illnesses, heart disease, diabetes, lung cancer, stroke, epilepsy, allergic responses, and others they think may apply. For our analyses, we summed the number of perceived consequences where higher counts indicated greater severity of perceived health consequences (range of 0-8).

##### Source

Perceived sources of indoor air pollution risks were assessed by asking, “What do you think are some of the sources of pollution inside your home?” Participants were encouraged to check all of the potential sources from a list of 11 items, which included cooking, vacuuming, smoking, microwave oven, gas heating, fireplace, open windows, insulation, pets, refrigerator, or other. For our analyses, we summed the number of perceived sources where a higher count indicates greater severity of perceived risk (range of 0-11).

##### Mitigation

Perceptions about the number of possible avenues to mitigate risk were assessed by asking, “What do you think are effective ways to reduce your exposure to indoor air pollution?” Participants were encouraged to check all effective ways from a list of 10 items, which included installing a range hood, opening windows, closing windows, installing an air purifier, changing air filters, cleaning the house, smoking outside instead of inside, installing an air quality monitor, cleaning air filters, and others that they think may apply. For our analyses, we summed the number of perceived mitigation strategies where a higher count indicates a greater number of perceived avenues for reducing risk (range of 0-10).

##### Air Quality

Perceptions of indoor air quality was assessed by asking participants, “On average, how would you rate the air quality in your home?” where 1=very poor and 5=very good.

##### Confidence

Confidence in knowing what to do to mitigate risk was assessed by asking, “How confident are you that you will know what actions to take if you learned that your indoor air quality was poor?” where 1=not at all confident and 5=extremely confident.

##### Behavior

Behaviors related to improve indoor air quality was assessed by asking participants, “In the past 3 months, have you made any changes in your home to improve the air quality?” where 1=yes, I have; 2=not yet, but I plan to; and 3=no, I have not and do not plan to. For our analyses, we recoded affirmative responses (“yes, I have or not yet, but I plan to”) as 1, and unenthusiastic responses (“no, I have not and don’t plant to”) as 0.

##### Empowerment

We used Rogers et al’s [26] empowerment scale that includes five constructs: self-esteem and self-efficacy, power and powerlessness, community activism and autonomy, optimism and control over the future, and righteous anger. Participants indicated their agreement level (1=strongly disagree and 5=strongly agree) on:

- nine statements related to self-esteem and self-efficacy (eg, “I generally accomplish what I set out to do”)
- seven statements related to power and powerlessness (eg, “I feel powerless most of the time”)
- six statements related to community activism and autonomy (eg, “People have a right to make their own decisions, even if they are bad ones”)
- four statements related to optimism and control over the future (eg, “People are limited only by what they think is possible”)
- four statements related to righteous anger (eg, “Getting angry about something is often the first step toward changing it”)

We created an overall measure of empowerment by taking the average of all 27 items (Cronbach alpha=.86).

##### Creativity

Previous research suggests creativity is inextricably linked to learning and experimentation [27,28]. Hence, we wanted to be able to control for creativity in our analyses to gain a more accurate measure of the sensor’s influence on learning, perceptions, and actions. We used Kirton’s short [29] Adaptation-Innovation Inventory where people rated their agreement (1=strongly disagree; 5=strongly agree) with statements describing themselves, such as “When involved in a project, I forget that other people are involved and should be consulted.” We created an overall measure of innovativeness by taking the mean of all 9 items (Cronbach alpha=.60).

#### Recruitment

Participants from the Pittsburgh area were recruited using Amazon’s Mechanical Turk, a Web-based survey platform [30-32], in spring and summer 2016. Participants (n=214) were invited to take a Web-based survey on air quality and were compensated US $1 for the 10- to 15-min survey. Participants (n=62) were also recruited from the local branches of the Carnegie Library of Pittsburgh that had Speck sensor indoor air monitors in their catalogs, made available courtesy of Carnegie Mellon University’s Community Robotics, Education and Technology Empowerment Lab. These participants were entered into a lottery for the chance to win 1 of 5 Speck sensors in exchange for their participation, and they completed the presurvey at one of the computer stations located in the library. At the time of recruitment, they were also informed of a follow-up survey that they would be invited to take after returning the Speck sensor (see study 2 for more details).

#### Participants

Participants reported being on average 36.2 years old (SD 12.26), with 55.6% (149/268) being female, 78.0% (206/264) having at least a college degree, and 44.7% (118/264) with a household income of US $51,000 or greater per year. Most identified as Democrats (119/264, 45.1%), followed by Independents (70/264, 26.5%), Republicans (46/264, 17.4%), Other (14/264, 5.3%), or Prefer Not to Answer (15/264, 5.7%). Most households had at least one child under the age of 18 years living at home (230/267, 86.1%), and of those, 9.0% (24/267) had at least one child under the age of 5 years. Most households also had at least one adult over the age of 65 years living at home (212/264, 80.3%), suggesting that many households were multigenerational. About 21.3% (56/263) of our participants reported that they or someone in their household suffered from a respiratory illness. Overall, the average long-term outdoor PM_2.5_ levels experienced by our participants were good (mean 10.48, median 9.97, SD 1.85). Of note, the Environmental Protection Agency’s federal long-term (annual average) standard is 15 μg/m^3^ and short-term (24-hour average) standard is 35 μg/m^3^ [33].

#### Data Analytic Plan

Statistical analyses were conducted using Stata version 14 (Stata Corp, College Station, TX, USA). One-sample *t* tests were used to assess whether self-reported knowledge, views on indoor air quality, and confidence in ability to improve air quality was different than average (midpoint test value of 3). Descriptive statistics were used to characterize views on health impacts, sources and mitigation options related to indoor air pollution, as well as for views on learning about indoor air quality. Logistic regressions were used to assess the following: (1) the consistency in people’s responses between sources of pollution and mitigation options, (2) the degree to which perceived home air quality and confidence in ability to improve poor quality predicted mitigation behavior, and (3) the extent to which intent to take action predicted interest in learning about air quality. All analyses controlled for empowerment and creativity where appropriate.

### Study 2: Views and Behaviors After Using Sensor

#### Survey and Interview Protocol

##### Survey

Participants checking out the sensor completed the first survey following the same protocol described in study 1. Upon returning the sensor to the library, participants were asked whether they would like to take a 10- to 15-min follow-up survey. They answered the same set of questions as before, with the addition of a few questions regarding their opinions. No compensation was offered for the follow-up survey.

##### Interview

Participants were asked about their views on indoor air pollution, managing indoor air pollution and the Speck sensor, as well as basic demographic questions.

#### Variables

The variables for study 2 were exactly the same as for study 1, with Cronbach alpha for empowerment being .89 and for creativity being .72.

#### Recruitment

##### Survey

Of the 62 participants who checked out the sensor and completed study 1, 26 agreed to participate in study 2 (attrition rate of 58.1%). Those who agreed to participate in study 2 did not meaningfully differ from those who elected not to participate, based on demographics, baseline knowledge, perceived home air quality, and confidence in ability to mitigate risk. Please refer to Multimedia Appendices 1 and 2 for more details.

##### Interview

Of the 62 participants who checked out the sensor and completed study 1, 4 agreed to be interviewed. The interviews lasted approximately 1 hour, were audio-recorded, and were transcribed for later analysis.

#### Participants

Participants reported being on average 44.5 years old (SD 12.6), with 61% (14/23) being female, 87% (20/23) having at least a college degree, and 57% (13/23) with a household income of US $51,000 or greater per year. Most identified as Democrats (11/23, 48%), followed by Independents (9/23, 39%), Republicans (1/23, 4%), or other (2/23, 9%). Many households had at least one child under the age of 18 years living at home (9/22, 41%), and of those, all (9/9, 100%) had at least one child under the age of 5 years. Few households also had at least one adult over the age of 65 years living at home (1/23, 4%). About 17% (4/23) of our participants reported that they or someone in their household suffered from a respiratory illness. Overall, the average long-term outdoor PM_2.5_ levels experienced by our participants were good (mean 10.56, median 10.36, SD 1.17).

#### Data Analytic Plan

One-sample *t* tests were used to assess whether participants saw the sensors as easy-to-use, accurate, or helpful for them to learn and if they would recommend or had recommended it to others. Paired-sample *t* tests were conducted to assess the impact of the sensor on self-reported air quality knowledge, perception of indoor air quality and confidence in ability to improve air quality, understanding of health impacts and sources of pollution, and knowledge of possible mitigation solutions. McNemar test was conducted to assess whether using the sensor resulted in people reporting having taken or intending to take mitigation measures to reduce risk, with a follow-up logistic regression to assess the association between, before, and after sensor mitigation behavior. Interview transcripts were coded for understanding of indoor air pollution, as well as beliefs and behaviors before and after using the sensor. Illustrative quotes and themes, including the percentage of the participants interviewed who mentioned them, are presented in the Results section. All analyses controlled for empowerment and creativity where appropriate.

## Results

### Study 1: Baseline Views and Behaviors of the General Public

#### What Do People Know and Do About Indoor Air Pollution?

In general, Pittsburgh residents reported knowing less than the average citizen (mean 2.62, SD 0.75) about indoor air quality (*t*_273_=−8.35, *P* ≤.001) (Table 1). Residents reported a median of 4 health consequences arising from indoor air pollution, with the most cited being asthma, allergic responses, lung cancer, and heart disease (Table 2). They also reported a median of 4 main sources contributing to indoor pollution, including pets, cooking, open windows, and gas heating. Residents saw a median of 6 actions as being most effective at reducing pollution, such as installing an air purifier, changing the air filter, cleaning the air filter, cleaning the house, and installing an air quality monitor. Logistic regressions found high internal consistency in reported sources and actions to mitigate risk. For example, those who reported that open windows contribute to air pollution were 8 times more likely to report closing windows mitigate risks (odds ratio [OR] 8.03, *P*<.001) and significantly less likely to report opening windows mitigate risks (OR 0.34, *P*<.001). However, there was one exception. Those who reported that vacuuming contributes to air pollution were 2 times more likely to report that cleaning is a way to reduce exposure (OR 2.12, *P*=.02). See Multimedia Appendices 3-5 for more details on internal consistency.

On balance, most people thought that their indoor air quality is relatively good (mean 3.31, SD 0.72, *t*_273_=18.69, *P*<.001) and were ambivalent about their confidence in knowing what to do should they learn their air quality was bad (mean 2.42, SD 0.96, *t*_275_=−1.44, *P*=.16) (Table 1). Despite this, most people reported that they had (56/276, 20.3%) or were intending to (122/276, 44.2%) take action to improve their indoor air quality. Moreover, a logistic regression found that those reporting better indoor air quality were significantly less likely to report having taken or intending to take future action (OR 0.65, *P*=.03), whereas those expressing greater confidence they would know how to mitigate being significantly more likely to have or to intend to take action (OR 1.69, *P*<.001). Whether those individuals actually have good air quality and if the actions taken effectively reduce the risk is unknown.

**Table 1:** One-sample *t* tests of knowledge, air quality, and confidence (midpoint of 3).

Variables	Mean (SD)	N	*t* statistic (degrees of freedom)	*P* value
Knowledge	2.62 (0.75)	274	−8.35 (273)	<.001
Air quality	3.31 (0.72)	274	7.15 (273)	<.001
Confidence	2.42 (0.96)	276	−10.1 (275)	<.001

**Table 2:** Percent of participants indicating possible health consequences, sources, and mitigation solutions related to indoor air pollution among the general public.

Survey prompts relating to indoor air quality knowledge		Participants who agreed, n (%)	
**Consequences**			
	Asthma		271 (100.0)
	Allergic responses		263 (97.0)
	Lung cancer		246 (90.8)
	Heart disease		130 (48.0)
	Stroke		68 (25.1)
	Epilepsy		45 (16.6)
	Diabetes		24 (8.9)
	Other		19 (7.0)
**Sources**			
	Pets		163 (60.1)
	Cooking		160 (59.0)
	Open windows		147 (54.2)
	Gas heating		140 (51.7)
	Vacuuming		127 (46.9)
	Insulation		111 (41.0)
	Fireplace		73 (26.9)
	Refrigerator		67 (24.7)
	Smoking		66 (24.4)
	Microwave oven		48 (17.7)
	Other		39 (14.4)
**Mitigation**			
	Installing air purifier		239 (88.2)
	Changing air filter		238 (87.8)
	Cleaning air filter		231 (85.2)
	Cleaning the house		217 (80.1)
	Installing air quality monitor		201 (74.2)
	Smoking outside instead of inside		151 (57.9)
	Installing range hood		131 (48.3)
	Opening windows		120 (44.3)
	Closing windows		75 (27.7)
	Other		14 (5.2)

#### Do People Want to Know More About Indoor Air Pollution?

Among those who are not already interested (checked the sensor out of the library), most people report wanting to know whether their indoor air quality is good or bad (146/195, 74.9%), with those claiming that they would indeed take action being those expressing the most interest in knowing about it (beta=.76, *P*<.001). These residents overwhelmingly preferred to learn about their indoor air quality through the use of an indoor monitor (152/195, 77.9%), followed by a local expert (101/195, 51.7%), social media (84/195, 43.1%), friends/family (67/195, 34.5%), flyers (60/195, 30.8%), community meetings (45/195, 23.1%), librarian (17/195, 8.7%), or other ways (15/195, 7.7%). People were even willing to pay for such a device, although at a price point (mean US $63.59, SD US $44.17) much lower than currently available monitors, which typically start at US $135 [34]. Residents were also interested in renting out a Speck monitor for free for a short period of time, with the two most convenient locations being work (119/195, 61.0%) and the public library (105/195, 53.8%).

### Study 2: Views and Behaviors After Using Sensor

#### How Do People View the Sensor?

On balance, interviewed participants reported being interested in using the sensor because of health concerns (4/4, 100%), curiosity (3/4, 75%), and its free availability at the library (1/4, 25%). In general, survey participants viewed the sensor quite favorably. Participants thought that the sensor was more easy to use (mean 4.24, SD 0.97, *t*_24_=6.39, *P*<.001) and accurate than average (mean 4.21, SD 0.72, *t*_23_=8.21, *P*<.001) (Table 3). They also reported that they felt like they learned from using the sensor (mean 4.12, SD 1.01, *t*_24_=5.53, *P*<.001) and would recommend or had recommended the sensor to others (mean 3.80, SD 1.15, *t*_24_=3.46, *P*=.01).

#### Does Using a Sensor Change What People Know and Do About Indoor Air Pollution?

A paired *t* test found that participants reported being more knowledgeable about indoor air pollution after using the sensor than they were before, (after: mean 2.77, SD 0.71; before: mean 2.38, SD 0.75; *t*_25_=−2.61, *P*=.02) (Table 4). Participants (3/4, 75%) we interviewed described having “a-ha moment[s]” (Participant K) when using the sensor where they felt like they learned something new about sources of indoor pollution:

...like, running the vacuum and cooking, and you know, things like that.

Participant G

After using the sensor, participants attributed indoor air pollution to biological (3/4, 75%), chemical (3/4, 75%), combustion (4/4, 100%), and dust/dander (3/4, 75%) sources and saw it as being much worse in the spring/summer (1/4, 25%) than at other times of the year.

**Figure 2:**
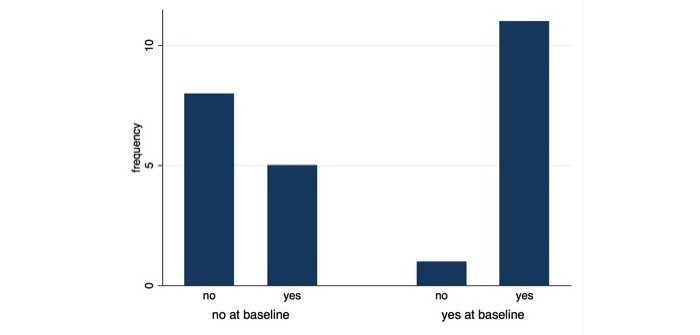
Reported or intended mitigation action after using the sensor for those who did not take or intend to take previous action before using the sensor (no at baseline) and those who did (yes at baseline).

Although we did not observe a significant difference in reported action, our findings suggest a trend toward taking or intending to take action to reduce indoor air pollution after using the sensor (McNemar χ^2^_1_=2.7 *P*=.10) (Figure 2). We also found those who reported having taken or intending to take action to mitigate their risk were significantly more likely to do so in the future (OR 17.6, *P*=.02). Indeed people reported that they had significantly better indoor air quality after using the sensor than before (after: mean 3.65, SD 0.75; before: mean 2.96, SD 0.77; *t*_25_=−5.20, *P*<.001), possibly as a result of what they did in response to what they learned. Participants we interviewed reported experimenting with the sensor (4/4, 100%), saying that they:

...moved [the sensor] around and tested various behaviors to see if it had any impact [on PMreadings].

Participant J

It was through this experimentation that participants discovered the impact of cooking (4/4, 100%), movement (2/4, 50%), and vacuuming (3/4, 75%) on indoor air pollution. They also used the sensor to monitor particulate levels in spaces such as their child’s room (Participant G) (3/4, 75%), where vulnerable people spend a lot of time, to make sure that air quality remained good.

We found that people felt more confident about knowing what to do to mitigate their risk after using the sensor (after: mean 2.62, SD 0.94; before: mean 2.31, SD 1.01; *t*_25_=−1.87, *P*=.07).

We observed no difference in mean number of reported sources of indoor air pollution and ways to mitigate risk. However, participants we interviewed reported taking new measures they had not tried before to reduce their exposure to indoor air pollution. These included improved pet care and maintenance (1/4, 25%) to reduce dander, a new furnace (1/4, 25%), cleaning more frequently and thoroughly (1/4, 25%), opening windows when cooking (1/4, 25%), and running ventilation systems when necessary (3/4, 75%). Our participants also expressed more concern about the consequences of indoor air pollution after using the sensor than before (after: mean 4.64, SD 1.66; before: mean 3.88, SD 1.56; *t*_24_=−2.10, *P*=.05) and seemed especially concerned about allergic responses, lung cancer, heart disease, and stroke (Table 5) with people wondering:

I have asthma...how can I improve my own air quality to avoid having an asthma attack?

Participant K

**Table 3:** One-sample *t* tests of views of the sensor (midpoint of 3).

Variables	Mean (SD)	N	*t* statistic (degrees of freedom)	*P* value
Easy	4.24 (0.97)	25	6.39 (24)	<.001
Accurate	4.21 (0.72)	24	8.21 (23)	<.001
Learn	4.12 (1.01)	25	5.53 (24)	<.001
Mitigation	3.2 (1.15)	25	.87 (24)	.40
Recommendation	3.8 (1.15)	25	3.46 (24)	.01

**Table 4:** Paired-sample *t* tests of knowledge, air quality, and confidence.

Variables	Before, mean (SD)	After, mean (SD)	N	*t* statistic (degrees of freedom)	*P* value
Knowledge	2.38 (0.75)	2.77 (0.71)	26	−2.61 (25)	.02
Air quality	2.96 (0.77)	3.65 (0.75)	26	−5.2 (25)	<.001
Confidence	2.31 (1.01)	2.62 (0.94)	26	−1.87 (25)	.07

However, not everyone made a change since they found they did not really need to do anything because their indoor air quality was not bad. As a result, they would not adopt any new measures (2/4, 50%) and moreover, one participant said:

I had no idea what I would do if it said it was bad [laughs].

Participant L

Participants also mentioned a number of barriers to reducing exposure, should the air quality be bad, such as lack of equipment (2/4, 50%), nearby polluters they have no control over (2/4, 50%), and pollution naturally being worse at certain times of year (2/4, 50%). For example:

We don’t have central air...if it’s hot, we need to have the window open.

Participant L

The sensor was also used outside of the home to help participants learn about their indoor air pollution in other settings (2/4, 25%):

I took it into work so it’s there.

Participant G

Indeed, one participant was able to use the output from the sensor to pressure building owners to make changes to improve the indoor air quality at work:

The office is right beside a nail salon and they were getting some really powerful smells and so they’re getting on the landlord about “something’s got to give, you know?” My folks can’t suffer like that, so I mean one of the things I’ve been—to be honest with you—one of the bargaining chips was, “well, listen we’re bringing this air monitor up so you’d better get your shit together...,” so they did, and we took the readings up there and they were generally pretty good.

Participant G

Not only did participants bring the sensor to places outside of the home, they also talked to other people about the sensor, encouraging them to use it (3/4, 75%):

I did tell my parents who live near me that they should check it out and see what their quality looks like.

Participant K

They also showed other people how to use it (1/4, 25%) and shared what they had learned about air pollution with others (1/4, 25%):

Some of the things we have learned just by seeing them...[I] would like to try to pass it on.

Participant G

**Table 5:** Percent of participants indicating possible health consequences, sources, and mitigation solutions related to indoor air pollution among the sensor users.

Survey prompts relating to indoor air quality knowledge		Participants who agreed, n (%)
**Allergic responses**		26 (100)
	Lung cancer	25 (96)
	Heart disease	17 (65)
	Stroke	10 (39)
	Epilepsy	8 (31)
	Diabetes	4 (15)
	Other	4 (15)
	Asthma	1 (4)
**Sources**		
	Cooking	21 (81)
	Vacuuming	18 (69)
	Open windows	15 (58)
	Pets	14 (54)
	Gas heating	14 (54)
	Refrigerator	5 (19)
	Microwave oven	5 (19)
	Other	5 (19)
	Smoking	4 (15)
	Insulation	3 (12)
	Fireplace	2 (8)
**Mitigation**		
	Cleaning the house	24 (92)
	Changing air filter	23 (89)
	Installing air purifier	22 (85)
	Cleaning air filter	20 (77)
	Installing range hood	18 (69)
	Installing air quality monitor	13 (50)
	Opening windows	12 (44)
	Smoking outside instead of inside	11 (42)
	Closing windows	8 (31)
	Other	2 (8)

## Discussion

### Principal Findings

In general, most people see themselves as knowledgeable about indoor air pollution, the sources of the pollution, and ways to mitigate their risk should they learn that their indoor air quality is poor. Although people report that they believe they have fairly good indoor air quality, they are not completely certain and are generally open to learning about it through the use of a portable indoor air quality monitor. People are willing to pay for such a monitor providing them with information about indoor air quality; however, the amount they are willing to spend is considerably less than that of those currently available. Therefore, making these monitors freely available to the public at a place that is convenient for them, such as at their local public library, is a way to help people access needed tools for informed decision making about indoor air quality.

We found that after using the sensor people reported higher levels of knowledge about indoor air pollution, confidence in their ability to improve indoor air, and improved indoor air quality (possibly as a result of taking mitigation actions). Moreover, we found a significant increase in the number of perceived health impacts after using the sensor, suggesting enhanced perceptions of risk. We also found a positive trend in action-taking among those who already took action before using the sensor *and* those who did not take action (and did not intend to do so in the future), suggesting the potential for this type of personalized risk information as an important motivating factor in prohealth behavior change.

Our findings also suggest that using the sensor was an interactive experience, where participants learned about the link between what they do in their home and what their exposure levels are. There is evidence that this type of experiential learning may be a more powerful way of helping people master new information and suggests a way to enhance motivation to make positive behavior changes [14,15]. These changes seemingly may have both a direct (people making changes in their own homes) and an indirect impact (people talking to others about it or making changes at their place of work [35,36]) on exposure levels, suggesting the potential for a positive ripple effect from using such a personalized device. Research looking at these direct and indirect impacts could be instructive to learn about the true potential and limitations of such monitors on reducing exposure to indoor air pollutants.

### Limitations

Although our study has very strong external validity, it is not without its limitations. First, we did not recruit a representative sample of Pittsburgh residents to participate in either study 1 or study 2, and therefore we cannot generalize our findings. However, we were mostly interested in evaluating those individuals most likely to use an indoor air quality monitor when made freely available. Future studies could be conducted to more rigorously evaluate the effect of using such monitors through a randomized controlled trial, allowing for more generalizable findings and a more thorough examination of underlying predictive factors.

Second, we were not able to collect actual exposure data since it was logistically difficult to offload data in real time from every single Speck checked out from a library branch and because of data privacy concerns. However, in this study we were less interested in actual exposure level and more interested in how the information induced changes in perceptions and self-reported behavior. A future study could look at actual PM_2.5_ levels along with knowledge and behaviors as predictors or covariates to better understand the relationship between these factors and outcomes.

Third, we did not ask our participants in study 2 what actions they took to improve their indoor air quality, nor did we ask them or evaluate which features of the sensor they found to be most persuasive in pursuing the given actions. Due to the design of our study, responses would likely have been subject to recall bias; thus, we did not pursue these lines of questions. However, a future study could ask participants to keep a running log of their activities and changes in behavior (with rationale) related to engagement with the sensor.

Fourth, given our design, we do not know whether learning one time from using the sensor is enough to influence actions over the long term. A future longitudinal study could help determine whether this type of short-term learning can lead to long-term impacts.

Finally, only a small subset of individuals who checked out the sensor from the library agreed to participate in a phone interview for our study. One possible reason is that email-based recruitment from a small sample for a time-consuming activity—with interviews lasting approximately 1 hour in length—is challenging and usually does not yield large numbers. Nonetheless, the researchers believe that the information gathered from the interviews that were conducted does yield insights into people’s perceptions and behaviors. Future studies should collect this valuable qualitative data that allow for deeper understanding of people’s views and actions.

### Conclusions

There is much to be hopeful about in these findings. Providing people with low- or no-cost portable indoor air quality monitors with a supporting Web-based platform that offers information about how to reduce risk can help people better express perceptions and adopt behaviors commensurate with the risks they face. Moreover, there appear to be other benefits from engaging in information about indoor air pollution through this experiential means, such as talking to others about the potential risks they may face and using the technology to make positive changes in indoor spaces other than the home. The emerging picture is that thoughtfully and well-designed personal sensor technologies can empower people to take control of the risks that they face and affect positive outcomes in their lives.

## Appendix

Multimedia Appendix 1 Comparison of the demographics of library patron participants who elected to participate in study 2 versus those who did not.

Multimedia Appendix 2 Comparison of knowledge, air quality, and confidence of library patron participants who elected to participate in study 2 versus those who did not.

Multimedia Appendix 3 Logistic regression evaluating whether opening or closing windows as a solution to poor indoor air quality predicts open windows as a source of pollution.

Multimedia Appendix 4 Logistic regression evaluating whether installing a range hood as a solution to poor indoor air quality predicts cooking as a source of pollution.

Multimedia Appendix 5 Logistic regression evaluating whether smoking outside as a solution to poor indoor air quality predicts smoking indoors as a source of pollution.

Multimedia Appendix 6 Logistic regression evaluating whether cleaning as a solution to poor indoor air quality predicts vacuuming as a source of pollution.

## Abbreviations

OR: odds ratio
PM _2.5_: particulate matter smaller than 2.5 microns
