# Supplementary material for: Effect of Using an Indoor Air Quality Sensor on Perceptions of and Behaviors Toward Air Pollution (Pittsburgh Empowerment Library Study): Online Survey and Interviews
Source: JMIR Mhealth Uhealth. 2018 Mar 8;6(3):e48. doi: 10.2196/mhealth.8273 (PMC5864999; doi:10.2196/mhealth.8273)
Supplement: Multimedia Appendix 1 [file mhealth_v6i3e48_app1.pdf]

	Agreed to participate	Did not agree to participate
	Mean (SD)	Mean (SD)
Age	44.5 (12.5)	45.4 (14.9)
Average long-term $PM_{2.5}$	10.56 (1.17)	10.46 (1.44)
	**%**	**%**
Female	60.9	55.7
At least a college degree	87.0	88.3
Political affiliation		
Democrat	47.8	45.0
Independent	39.1	25.0
Republican	4.4	5.0
Other	8.7	25.0
Children		
At least one under 18	40.9	39.3
Of those, at least one under 5	40.9	39.3
At least one adult over 65	4.4	13.3
Respiratory illness	17.4	21.7
