# Supplementary material for: Effect of Using an Indoor Air Quality Sensor on Perceptions of and Behaviors Toward Air Pollution (Pittsburgh Empowerment Library Study): Online Survey and Interviews
Source: JMIR Mhealth Uhealth. 2018 Mar 8;6(3):e48. doi: 10.2196/mhealth.8273 (PMC5864999; doi:10.2196/mhealth.8273)
Supplement: Multimedia Appendix 2 [file mhealth_v6i3e48_app2.pdf]

Variable	Agreed Mean (SD)	Did not agree Mean (SD)	F	*P*-value
Knowledge	2.37 (.74)	2.77 (.91)	3.46	.07
Air quality	2.96 (.76)	3.14 (.60)	1.09	.30
Confidence	2.30 (.99)	2.58 (1.18)	1.04	.31
