# Supplementary material for: Effect of Using an Indoor Air Quality Sensor on Perceptions of and Behaviors Toward Air Pollution (Pittsburgh Empowerment Library Study): Online Survey and Interviews
Source: JMIR Mhealth Uhealth. 2018 Mar 8;6(3):e48. doi: 10.2196/mhealth.8273 (PMC5864999; doi:10.2196/mhealth.8273)
Supplement: Multimedia Appendix 3 [file mhealth_v6i3e48_app3.pdf]

Source	n	odds ratio	s.e.	*P*-value
Opening windows	267	.34	.09	.001
Closing windows	267	8.03	2.88	.001
