# Supplementary material for: Effect of Using an Indoor Air Quality Sensor on Perceptions of and Behaviors Toward Air Pollution (Pittsburgh Empowerment Library Study): Online Survey and Interviews
Source: JMIR Mhealth Uhealth. 2018 Mar 8;6(3):e48. doi: 10.2196/mhealth.8273 (PMC5864999; doi:10.2196/mhealth.8273)
Supplement: Multimedia Appendix 4 [file mhealth_v6i3e48_app4.pdf]

| Source | n | odds ratio | s.e. | *P*-value |
|---|---|---|---|---|
| Cooking | 267 | .84 | .25 | .56 |
