# Supplementary material for: Effect of Using an Indoor Air Quality Sensor on Perceptions of and Behaviors Toward Air Pollution (Pittsburgh Empowerment Library Study): Online Survey and Interviews
Source: JMIR Mhealth Uhealth. 2018 Mar 8;6(3):e48. doi: 10.2196/mhealth.8273 (PMC5864999; doi:10.2196/mhealth.8273)
Supplement: Multimedia Appendix 5 [file mhealth_v6i3e48_app5.pdf]

Source	n	odds ratio	s.e.	*P*-value
Smoking inside	267	1.01	.26	.87
